# Moral Attitudes Toward Pharmacological Cognitive Enhancement (PCE): Differences and Similarities Among Germans With and Without PCE Experience

**DOI:** 10.3389/fphar.2018.01451

**Published:** 2018-12-10

**Authors:** Sabine Pohl, Hannes Boelsen, Elisabeth Hildt

**Affiliations:** ^1^Department of Philosophy, University of Education Karlsruhe, Karlsruhe, Germany; ^2^Department of Philosophy, Johannes Gutenberg University Mainz, Mainz, Germany; ^3^Center for the Study of Ethics in the Professions, Illinois Institute of Technology, Chicago, IL, United States

**Keywords:** autonomy, distortion of competition, ethics, fairness, interview study, pharmacological cognitive enhancement, stimulants

## Abstract

Pharmacological cognitive enhancement (PCE), the use of illicit and/or prescription drugs to increase cognitive performance, has spurred controversial discussion in bioethics. In a semi-structured interview study with 60 German university students and employees, differences and similarities in moral attitudes toward PCE among 30 experienced participants (EPs) vs. 30 inexperienced participants (IPs) were investigated. Substances EPs used most often are methylphenidate, amphetamines, tetrahydrocannabinol and modafinil. Both EPs and IPs addressed topics such as autonomous decision making or issues related to fairness such as equality in test evaluation and distortion of competition. While most EPs and IPs were convinced that the decision of whether or not to use PCE is part of their individual freedom, their views varied considerably with regard to fairness. IPs considered issues related to fairness as much more critical than EPs. Thus, a person’s moral attitudes toward PCE may not only depend on moral common sense, but also on whether they have used illegal and/or prescription drugs for PCE before. This points to the importance of including the various relevant stakeholder perspectives in debates on the ethical and social implications of PCE.

## Introduction

Pharmacological cognitive enhancement (PCE), the use of illicit substances and/or prescription drugs (off-label use) to improve cognitive performance, has become an often-discussed topic in bioethics over the last decade (Schelle et al., 2014: p. 1; Garasic and Lavazza, 2016: p. 1; Schleim and Quednow, 2018). The use of PCEs in students and employees has been reported in several German surveys (Dietz, 2011; Franke et al., 2013; DAK, 2015). In general, the off-label use of prescription drugs and/or the use of illicit drugs is considered more problematic than the use of coffee or over the counter drugs such as caffeine, ginkgo or nicotine. Substances that have been consumed for PCE are psychostimulants, antidementia drugs, and antidepressants, among others (Metzinger, 2012: 38; see, for example, Mache et al., 2012; Franke et al., 2014). Besides the discussion whether these substances have any objective detectable cognition enhancing effects on users (Repantis et al., 2010: p. 475–480; Farah et al., 2014: p. 97–100), there are normative concerns on the use of PCE (Schelle et al., 2014; Faber et al., 2016; Garasic and Lavazza, 2016; O’Connor and Nagel, 2017). A number of studies have investigated attitudes toward PCE (Babcock and Byrne, 2000; Franke et al., 2012a,b; Partridge et al., 2012; Bell et al., 2013; Sattler et al., 2013; Vrecko, 2013; Singh et al., 2014; Maier et al., 2015; Ram et al., 2017; Vagwala et al., 2017).

The aim of this interview study was to find out what moral attitudes toward pharmacological enhancement participants with PCE experience (experienced participants, EPs) and without PCE experience (inexperienced participants, IPs) are aware of and whether those vary between the two groups. In this, the term “moral attitude” refers to the moral “evaluation of an object, concept, or behavior along a dimension of favor or disfavor, good or bad, like or dislike” (Ajzen and Fishbein, 2000: p. 3). Here, we focus on autonomy, fairness related aspects (equality in test evaluation and distortion of competition) and motivations to use PCE. The findings give an insight into the differences between the views of EPs and IPs by directly comparing them.

## Methods

Semi-structured interviews were performed at the University of Mainz in 2015–2016. From January 2015 on, following approval from the local Ethics Committee of the Landesaerztekammer Rhineland-Palatinate, participants were recruited by flyers posted in public places such as the university, supermarkets, pharmacies and local firms, on the internet, and with support of the Gutenberg Brain Study in Mainz. Incentives were offered for participation (in the first phase of the recruitment 25€, in the second phase 50€). The recruitment process received a limited response, and more students responded than other groups. We explain this by the sensitivity of the topic, especially in professional context. We interviewed 60 people. 30 were experienced with PCE, 30 were not. Participants were aged 18–45 years (mean age = 26,93, *SD* = 6,11), 45% [*n* = 27] were female, 68,3% [*n* = 41] were students. Substances used by EPs were methylphenidate (*n* = 21), (meth-)amphetamines (*n* = 14), tetrahydrocannabinol (*n* = 3), modafinil (*n* = 2), anti-dementia drugs (*n* = 1), ß-blockers (*n* = 1), cocaine (*n* = 1), ephedrine (*n* = 1). Some EPs (*n* = 8) used more than one substance for PCE.

Experienced participants and IPs were matched according to age and gender. By EP, people were categorized who have used or still were using illicit substances and/or prescription drugs (off-label use) for cognitive enhancement. By IP, people were categorized who have never used illicit substances and/or prescription drugs (off-label use) for PCE. The use of illicit drugs exclusively in a spare time context did not lead to an exclusion from the IP group, since PCE was defined as substance use for cognitive enhancement only. To exclude self-medication, diagnoses of psychiatric disorders treated with substances that also could be used for PCE, led to an exclusion from the study. A psychological SCID Interview (Structured Clinical Interview for DSM-IV) was conducted with the participants first. Persons diagnosed with potential anxiety disorders or other during the SCID Interview were excluded from the study. People who had been diagnosed with attention deficit hyperactivity disorder (ADHD), depression or anxiety disorder in their medical history were also excluded. Lifetime diagnoses were hypothesized to avoid the possibility of self-medication. Although it is possible that people treated with prescription drugs modify the doses to enhance their performance, it would have been almost impossible for us to decide whether this was treatment or enhancement. For this reason, we excluded persons with the above-named diagnoses.

Five interviewers were trained to interview the participants. The interviews took place in a separate room with one interviewer talking to one participant. They were recorded with voice recorders and transcribed, all data was pseudonymized. The transcribed interviews were coded independently by SP and HB with MAXQDA 12.0.2, according to the Grounded Theory approach (Strauss and Corbin, 1990; Bryant and Charmaz, 2010). Differences in coding were discussed and agreed upon in open discourse. This was necessary in rare cases when answers were imprecise. The aspects presented in this paper were coded uniformly by both coders.

Concerning moral aspects of PCE use, the following open questions were asked: Do you think there are general reasons speaking for the use of PCE? Do you think there are ethical or moral reasons speaking for the use of PCE? Do you think there are general reasons speaking against PCE? Do you think there are ethical or moral reasons speaking against PCE? Do you think the use of PCE is fair? Does PCE lead to distortion of competition? Questions were open to avoid bias in the participants answers.

## Attitudes Toward Autonomous Decision-Making and Fairness

### Autonomous Decision-Making

According to Christman (2015), the concept of autonomy is “generally understood to refer to the capacity to be one’s own person, to live one’s life according to reasons and motives that are taken as one’s own and not the product of manipulative or distorting external forces.” Amongst other aspects, it “concerns the freedom to decide what to believe in and to weigh the pros and cons of a given course of action” (Garasic and Lavazza, 2016: p. 4). Following this understanding, participants’ statements concerning whether or not individuals should be free to use PCE were categorized as statements concerning *autonomous decision-making*.

Almost two-thirds of the participants [*n* = 37] came up with autonomy-related aspects. 11 EPs and 14 in the IP group claimed people should decide autonomously to use/disuse PCE. In either group only one participant did not agree.

A typical EP opinion was:

“I think the moral aspect is massively overrated, in the end it is a question everyone has to decide for themselves. It is part of individual freedom to use such possibilities. After, of course, having dealt with the risks.”

Experienced participants typically argued in favor of autonomous decision-making: Individuals should be free to decide whether they want to use PCE to enhance their performance. They argued individuals should inform themselves about risks related to PCE, such as side effects or risks of addiction. Most of the IPs shared the EPs’ opinion that everyone should be free to use or disuse PCE.

A typical IP opinion was:

“[A]s long as I do not harm anyone but myself, [...] I believe everyone should consume, what they want. Also marihuana and, for all I care, even Ritalin, if they need it once in a while. I guess everyone knows, has to know, what they are doing to their body.”

Inexperienced participants highlighted the necessity of being well informed. Users then would have to calculate risks and expected benefits. Afterward they could decide if they were willing to take the risks that might possibly occur.

### Fairness

Statements concerning fairness, equality, and distortion of competition were categorized under *fairness*. Almost two-thirds of the EPs [*n* = 18] did not think it was unfair to use PCE to enhance their performance, few [*n* = 2] found it rather not unfair. Some [*n* = 4] said, it was unfair, few [*n* = 2] said it was rather unfair. Few EPs [*n* = 3] thought that fairness was not even a factor in PCE for they did not believe being in a competition. IPs’ views on fairness were contrary: Almost half of them [*n* = 13] thought of PCE as unfair, only few [*n* = 3] said it was not unfair. Some [*n* = 4] were undecided, one fifth [*n* = 6] did not comment. EPs were more likely to consider their behavior as not unfair, whereas most IPs shared the view that PCE was unfair.

In respect of the difficulty of clarifying the various meanings of fairness (Schelle et al., 2014: p. 8), findings were divided into different aspects of fairness: *equality in test evaluation* and *distortion of competition*.

A typical EP answer was:

“I think, it’s not [unfair], because everyone has the choice, and because I am not better than someone who deserved their top grade, I never got the top grade with it [using PCE]. I would say, it just helps me to deal with this monster called revision and exams better.”

With regard to the achievements that can be made with support of PCE, this EP argued that only one defined best grade can be realized in an exam. Since nobody could do better, the EP believed PCE not to be unfair. Some of the EPs said they used PCE primarily to get through the learning phase and to pass the test:

“When I am in an exam situation […], then I don’t take the exam to be better than the others, but to show that I studied and, of course, to pass the exam. I don’t think I am in competition with others.”

They do not think PCE is unfair because they do not use it to outperform others, but to cope with learning struggles.

IPs saw this differently:

“It’s unfair in the sense that […] you are better than the others, better than your peers.”

Other than the EPs, most IPs believed that users would perform better than those who did not use performance enhancers. In consequence, they were convinced it was unfair to use PCE in competitive situations.

Opinions on noticeable effects of PCE on users’ performance that negatively influence conditions in competitive contexts were categorized as *distortion of competition*. Almost half of the EPs [*n* = 14] believed that there was no distortion of competition between users and non-users in test situations, some [*n* = 8] believed, it was. In contrast, almost half of the IPs [*n* = 14] said, distortion of competition was given if users and non-users were compared to each other, only a few [*n* = 3] did not think so.

Some participants referred to differences in individuals’ mental abilities and believed enhancement could lead to more equality by closing the gap between people with lower and higher cognitive abilities. One EP said too few people were using enhancement to seriously distort competition in society.

Some IPs shared the opinion that PCE does not lead to distortion of competition because users had to recover from using PCE:

“I don’t think [it is a distortion of competition] because it will balance out overall. The people who study for 3 days in a row then have to sleep for 3 days afterward. I think they just shoot themselves in the foot with it.”

However, typically IPs in this study believed distortion of competition was caused, assuming users were in a better position competitively than those not using PCE:

“[I]f you really use it for enhancement, then the performance will accordingly be better. [...] Not, because the person is really better, but because they took the stimulant drug in that moment.”

They were convinced PCE had noticeable effects that lead to better performance in test situations.

## Agreeing on Autonomy, Disagreeing on Fairness

Most of the participants mentioned autonomy-related aspects and thought that individuals should be free to decide on whether or not to use PCE substances, similar to the results of Bell et al. (2013) and Forlini and Racine (2009), but in contrast to a prior interview study (Franke et al., 2012b) in which aspects related to autonomous decision-making did not play an essential role. How can the differences in fairness-related moral attitudes be explained, if EPs and IPs think that autonomy is important?

According to the interviews, EPs aimed for enhancing their learning skills, staying awake longer or just passing a test or exam. None of them aimed to enhance their abilities beyond normal human capabilities. Most of them used PCE whilst learning. They perceived a positive effect using PCE substances but seemed to believe this effect only has an impact on their own performance and was not a disadvantage for third persons. When it comes to fairness, a clear majority of the EPs said it was not unfair to enhance performance with PCE substances, whereas a majority of IPs said it was unfair. This discrepancy could possibly be explained by the EPs’ belief that using PCE does not affect others: If there are no effects on third parties, there is no moral reason to limit one’s autonomy. Another possible interpretation for these findings is that PCE can be considered a procedure people undergo who want to keep up with their environment and who do not want to be left behind in certain situations (Franke et al., 2012a; Sattler et al., 2013; Forlini and Hall, 2016: p. 3).

Most IPs, on the other hand, were convinced that there was a detectable effect on the results of tests or likewise and therefore believed distortion of competition takes place. Coherently they had the opinion that it is unfair to use substances in such competitive situations. The IPs’ views are in accordance with a study by Faber et al. (2016) who discussed the unacceptability of PCE in laypeople against the background of the Unfairness-Undeservingness Model and the Hollowness-Undeservingness Model: “The Unfairness-Undeservingness Model holds that people judge PCE to be unacceptable because they take it to produce unfairness and to undermine the degree to which PCE-users deserve reward. The Hollowness-Undeservingness Model assumes that people judge PCE to be unacceptable because they find achievements realized while using PCE hollow and undeserved.” (Faber et al., 2016: p. 1).

In addition, the views toward fairness expressed by EPs might be interpreted against the background of the use of prescription and/or illicit drugs for PCE not being commonly accepted in society. The awareness of a negative public attitude toward PCE may lead EPs to justify themselves for using PCE. A strategy like this can be seen as being in line with a framework presented by Shalvi et al. (2015), according to which self-serving justification processes may allow people to engage in moderate unethical behavior and still feel moral about it. If people can justify their behavior, they can bridge the gap between their own benefit gained by the action and their view on themselves as being moral persons. Accordingly, the different moral justifications of PCE in EPs and IPs in our study might be explained by the self-serving justification. Another possible explanation for the different views EPs and IPs had on aspects related to fairness might be that positive attitudes toward drugs do correlate with the use of the drug, as Schelle et al. (2014) summarize.

Furthermore, EPs in our study did not consider the positive effects of PCE as being very strong. Although PCE might help them focus better, they were aware that the drugs are not magic potion that leads to outstanding results without still having to work hard. In view of this, they did not perceive PCE as distorting competition. In contrast, the IPs seemed to assume the effects of PCE as being stronger than the EPs did. They believed that users had found an easier method for gaining better results than others who without PCE had to work harder for a good performance. There clearly is a difference in the perception EPs and IPs had of PCE effects. In fact, intra- and interpersonal effects of PCE substances have been reported to vary (Husain and Metha, 2011; Van der Schaaf et al., 2013). These variations may explain part of the disorientation concerning the effects of PCE, which may also influence moral perspectives on the topic.

Reasons given for not using PCE or for not using PCE any more were mainly health risks, in other words the fear of harming oneself. This is interesting, since users in general tend to rate PCE as less dangerous than non-users, as Eickenhorst et al. (2012) found. Taking into account that non-users rate the effects of PCE stronger than users, they might also believe the health risks are higher, and may not use PCE for that reason. To decide whether unwanted side effects are worth the benefits is part of the decision for or against the use of PCE (Maier and Schaub, 2015: p. 157). The main moral reason given to not use PCE substances is the fear of harming oneself and not the possible implications on third persons. This aspect has been discussed in several studies under the term “medical safety”: the more people were concerned about aspects of medical safety, the more they tended to object to substance use for PCE (Scheske and Schnall, 2012; Santoni de Sio et al., 2016).

To summarize, in our small sample size that does not allow generalization, fairness-related moral attitudes on PCE differed considerably depending on whether or not the interviewees were experienced with PCE. These results are in line with similar studies. In particular, IPs considered issues related to fairness as much more critical than EPs. Thus, one’s view on PCE might not only depend on moral common sense (like “using drugs is wrong,” “cheating is wrong”), but also on having or not having used illegal/prescription drugs for PCE before. While this may not seem totally surprising, it points to the importance of including the various relevant stakeholder groups in debates on the ethical and social implications of PCE. In addition, as EPs said their PCE use was primarily motivated by the wish to “keep up” with others at school/university or at work, it is advisable to further analyze working and studying conditions and how these conditions affect individual’s motivation and willingness to use illicit or prescription drugs for PCE.

## Author Contributions

EH, SP, and HB planned and developed the project, and wrote the sections of the manuscript. HB and SP interviewed the participants and analyzed the interview with the support of EH. SP wrote the first draft of the manuscript. All authors revised the manuscript, and read and approved the submitted version.

## Conflict of Interest Statement

The authors declare that the research was conducted in the absence of any commercial or financial relationships that could be construed as a potential conflict of interest.
